# Clinical Features and Polysomnographic Findings in Greek Male Patients with Obstructive Sleep Apnea Syndrome: Differences Regarding the Age

**DOI:** 10.1155/2012/324635

**Published:** 2012-01-23

**Authors:** Efremidis George, Varela Katerina, Spyropoulou Maria, Beroukas Lambros, Nikoloutsou Konstantina, Georgopoulos Dimitrios

**Affiliations:** Sleep Lab, 2nd Respiratory Department, South-Western Greece Chest Hospital, 26226 Patras, Greece

## Abstract

*Background-Aim*. Although sleep disturbance is a common complaint among patients of all ages, research suggests that older adults are particularly vulnerable. The aim of this retrospective study was to elucidate the influence of age on clinical characteristics and polysomnographic findings of obstructive sleep apnea syndrome (OSAS) between elderly and younger male patients in a Greek population. *Methods*. 697 male patients with OSAS were examined from December 2001 to August 2011. All subjects underwent an attended overnight polysomnography (PSG). They were divided into two groups: young and middle-aged (<65 years old) and elderly (≥65 years old). We evaluated the severity of OSAS, based on apnea-hypopnea index (AHI), and the duration of apnea-hypopnea events, the duration of hypoxemia during total sleep time (TST) and during REM and NREM sleep, and the oxygen saturation in REM and in NREM sleep. *Results*. PSG studies showed that elderly group had significant higher duration of apnea-hypopnea events, longer hypoxemia in TST and in NREM sleep, as well as lower oxygen saturation in REM and NREM sleep than the younger group. Otherwise, significant correlation between BMI and neck circumference with AHI was observed in both groups. *Conclusions*. The higher percentages of hypoxemia during sleep and longer duration of apnea-hypopnea events that were observed in the elderly group might be explained by increased propensity for pharyngeal collapse and increased deposition of parapharyngeal fat, which are associated with aging. Another factor that could explain these findings might be a decreased partial arterial pressure of oxygen (PaO_2_) due to age-related changes in the respiratory system.

## 1. Introduction

Obstructive sleep apnea syndrome (OSAS) is a highly prevalent disorder characterized by instability of the upper airway during sleep, which results in markedly reduced (hypopnea) or absent (apnea) airflow at the nose/mouth [1]. Episodes are typically accompanied by oxyhemoglobin desaturation and terminated by brief microarousals that result in sleep fragmentation [2]. Patients usually present with loud habitual snoring, witnessed apnea, and excessive daytime sleepiness [1]. Several physical and psychological changes are known to occur with normal aging. It is well known that there are differences of OSAS regarding the elderly [3]. Aging is associated with several well-described changes in patterns of sleep. Although sleep disturbance is a common complaint among patients of all ages, research suggests that older adults are particularly vulnerable [3]. Studies about the clinical characteristics of OSAS have been done mainly in middle-aged adults and there is little information on the differences in polysomnographic findings of OSAS between elderly and younger adults. A high prevalence of OSAS of 30.5% [4] up to 81% [3] was reported in the elderly (≥65 years old) when OSAS was defined as an apnea-hypopnea index (AHI) of more than 5 events per hour. Therefore, aging could be considered as a risk factor for the development of OSAS [5]. In addition, in previous studies, body mass index (BMI)—one of the most typical parameters which correlates significantly with AHI in young patients with OSAS—was not associated with OSAS in elderly patients [6]. The differences in clinical characteristics of OSAS between young or middle-aged and elderly patients are not fully explained.

## 2. Methods

### 2.1. Subjects

We enrolled 924 Greek male patients who were referred to our sleep laboratory, from December 2001 to August 2011. All subjects underwent an attended overnight polysomnography (PSG). 697 of them were found to have OSAS (AHI ≥ 5 events per hour of sleep), while COPD patients and patients with heart failure excluded. Patients were divided into two groups: young and middle-aged (<65 years old, *n* = 568) and elderly (≥65 years old, *n* = 129). The severity of OSAS was determined from the apnea-hypopnea index (AHI) for TST [2]. The patients completed a full report with their daytime and nocturnal symptoms. The principal daytime manifestation was excessive sleepiness but other symptoms, such as unrefreshing sleep, poor concentration, fatigue, and headache, were also reported. The most common nocturnal symptoms were snoring, choking, or gasping during sleep and recurrent awakening. In order to measure the subjective daytime sleepiness, all patients completed the Epworth Sleepiness Scale (ESS) [7]. The diagnosis of OSAS was based on the combination of these symptoms and the results of PSG [2].

### 2.2. Polysomnography (PSG)

All subjects underwent an attended overnight polysomnography (Alice 4, Sleepware; Phillips-Respironics, USA, and Somnoscreen-Domino 2.3.0; Somnomedics, Germany). Briefly, sleep state was recorded with two channels of EEG (C4/A1, C3/A2), two channels of electro-oculogram, and one-channel submental electromyogram. Breathing was assessed by monitoring chest wall and abdominal movements using strain gauges and nasal and oral flows using thermistors (until 2005) and nasal pressure transducers (after 2005). Arterial oxygen saturation was measured continuously by pulse oximetry, using a finger probe. Body position was assessed continuously with a body position sensor. Leg movements were monitored with two channels of electromyogram, and an ECG was recorded continuously. Sleep stages and respiratory events were scored manually according to the old “Chicago criteria” of American Academy of Sleep Medicine (AASM) until 2007 [2] and with the new criteria of AASM after 2007 [8]. Apnea was defined as reduction in airflow greater than ≥90% of baseline, which was lasting ≥10 sec and affecting at least the 90% of the event. Obstructive apnea was estimated when respiratory effort was recorded throughout the event, central apnea was estimated when respiratory effort was absent, and mixed apnea was estimated when respiratory effort was absent at the beginning of the event followed by increasing respiratory effort during the second half. Hypopnea was defined as reduction in airflow by ≥30% from baseline, which was lasting ≥10 sec, affecting at least 90% of the event, followed by reduction in saturation at least ≥4% from baseline SatO_2_% prior to the event. Alternatively, hypopnea was defined as a reduction in airflow ≥50% from baseline, which was lasting ≥10 sec, affecting at least 90% of the event followed by reduction in saturation ≥3% from baseline SatO_2_% prior to the event or appearance of an arousal [9]. Apnea-Hypopnea Index (AHI) was defined as the total number of episodes of apneas and hypopneas per hour of real sleep [8].

In addition, we measured AHI in REM and NREM sleep, mean duration of apnea and hypopnea events, duration of hypoxemia during TST and during REM and NREM sleep, and oxygen saturation in REM and in NREM sleep.

### 2.3. Statistical Analysis

SPSS version 14.0 for Windows (SPSS Inc., Chicago, Ill, USA) was used for the statistical analysis. The results are presented as means ± SD. The Kolmogorov-Smirnov test was used to confirm normality. Differences in clinical variables among the three groups were assessed by analysis of variance (ANOVA) followed by Tukey's post-hoc test or the Kruskal-Wallis test for continuous variables and the *χ* ^2^ test for categorical variables. Spearman's correlation coefficients were calculated to determine the relationships between AHI and obesity-related variables, and stepwise multiple regression analysis was used to identify factors that contributed to the severity of OSAS. A value of *P* < 0.05 was considered as the threshold for statistical significance.

## 3. Results

There were no significant differences in baseline characteristics such as BMI and neck circumference among the two groups (Table 1).

Also, there were no differences between two groups in relation with subjective characteristics and symptoms as daytime sleepiness, fatigue, headache, and so forth. Differences were observed in cardiac arrhythmias as they were the commonest in elderly patients, as it was expected (Table 1).

Findings obtained from PSG studies showed that there was no significant difference in AHI between the two groups (Table 2). Other findings revealed that the elderly group had significant higher mean duration of apnea-hypopnea events (*P* < 0.001) and longer duration of hypoxemia in TST (*P* = 0.004) and in NREM sleep (*P* = 0.021) than the younger group. Also they had significant lower oxygen saturation in REM (*P* = 0.013) and NREM sleep (*P* = 0.027) than younger patients (Table 2).

In both groups, there were significant correlations between obesity-related anthropometric variables and the severity of OSAS (value of AHI). So there was significant correlation between BMI with AHI in elderly group (*r* = 0.157, *P* = 0.026) and in the younger group (*r* = 0.326, *P* < 0.001, Table 3). Also, there were significant correlations between neck circumference and AHI in the above groups (*r* = 0.245, *P* < 0.001 and *r* = 0.380, *P* < 0.001, resp., Table 3).

In the stepwise multiple regression analysis of obesity-related variables, BMI and neck circumference were independently related with AHI in the elderly group (*r* = 0.043, *P* = 0.035 and *r* = 0.227, *P* = 0.001, resp.), as well as in the younger group (*r* = 0.012, *P* = 0.008 and *r* = 0.368, *P* < 0.001, resp., Table 3).

## 4. Discussion

In the present retrospective study, patients with OSAS were evaluated for about a decade ago. There were no significant differences in BMI and AHI between the two age groups, but neck circumference and BMI were the most significant determinants of AHI in all patients based on the stepwise multiple regression analysis.

BMI has been one of the most typical parameters in evaluating the relationship between OSAS and obesity [1, 10]. In this study, BMI was significantly related with AHI in both groups. A previous study reported that BMI and neck circumference were not associated with OSAS in elderly patients [6], but that study might be limited by small sample size. The higher prevalence of OSAS in elderly adults could be explained by other factors besides, obesity: decreases in ventilatory control, muscular endurance, and thyroid function, and increased upper-airway collapsibility and sleep fragmentation potentially contribute to the development of OSAS in the elderly [11]. In addition, only a few elderly patients (*n* = 11) with normal BMI (under 25 Kgr/m^2^) had OSAS. Therefore, obesity still plays an important role in patients with OSAS.

It is generally accepted that obesity characteristics like neck circumference is a major risk factor of OSAS [12]. In our study, neck circumference is the most independently related factor with AHI in all patients. It is also accepted that neck's circumference increasing is a factor for an increased parapharyngeal fat deposition [13, 14]. It is well known that aging increases the propensity for pharyngeal collapse [11, 13], as well as the deposition of parapharyngeal fat in the elderly [13], but the size of this parapharyngeal fat pad increases independently of body mass index [11, 13]. This age-related upper-airway collapsibility could also explain the increased prevalence of OSAS in elderly patients [11, 14]. Neck circumference in combination with a decreased reflex sensitivity in upper airways which increase with age [11, 15] may cause severe OSAS in the elderly in the present study.

Some studies have suggested that cardiac arrhythmias are common problems in OSAS patients, although the true prevalence and clinical relevance of cardiac arrhythmias remain to be determined [16]. There are many different types of arrhythmias linked to OSAS, such as atrial and ventricular premature extrasystoles, nonsustained ventricular tachycardia, sinus arrest, and second-degree atrioventricular conduction block, which are reported 30%–50% in patients with OSAS and increased with the number of apneic episodes and severity of the associated hypoxemia [17]. In our study, 20% of elderly patients suffer from cardiac arrhythmias.

In the present study, apnea/hypopnea duration and nocturnal hypoxemia duration with oxyhemoglobin saturation under 90% were longer in the elderly group compared to the other group. This nocturnal hypoxemia is more severe in NREM sleep, stage at which elderly patients sleep more [18]. With age, the percentage time of rapid eye movement (REM) sleep decreases, while the percentage of light sleep (stage 1 and stage 2 of NREM sleep) increases [18]. However, in the current theoretical framework, such changes in sleep architecture are considered nonpathological and might reflect age-related neural degeneration [19]. In addition, the adaptive potentialities of external respiration were found to be limited. Ware et al. [20] also reported that the duration of apnea events was longer in their elderly compared to middle-aged OSAS patients, and breakdown in sensing apnea events and reduction in the stimulus triggering arousals were speculated to affect the long duration of apnea events and therefore of hypoxemia duration in elderly patients. In addition, increased pharyngeal collapsibility with aging might also explain the longer duration of hypoxemia in our study as previously reported [13, 21, 22]. Also, aging is associated with a decrease in partial arterial pressure of oxygen (PaO_2_) due to age-related changes in the respiratory system [23]. Therefore, it is obvious that the degeneration of respiratory center and oxygen receptors due to age results in longer hypoxemia during sleep.

This retrospective study was performed based on the data from a real clinical setting with a relatively large sample size. Therefore, the differences in the clinical characteristics and in polysomnographic findings of OSAS between elderly and young or middle-aged patients could have clinical relevance. However, the patients were referred to the clinic for evaluation of possible sleep apnea. Thus, the results may have been influenced by referral bias and survivorship effects.

In conclusion, BMI and neck circumference still influence the severity of OSAS in elderly patients. It seems that aging increases the propensity for pharyngeal collapse, as well as the deposition of parapharyngeal fat in the elderly. Also, it is associated with a decrease in partial arterial pressure of oxygen (PaO_2_) due to age-related changes in the respiratory system (with degeneration of respiratory center and oxygen receptors), which results in longer hypoxemia during sleep. These structural and functional changes could explain higher percentages of hypoxemia (particularly in NREM sleep) and longer duration of apnea-hypopnea events were observed in the elderly group. Therefore, further studies are needed to determinate the causes of all these findings.

## Figures and Tables

**Table 1:** Demographic and clinical characteristics of Greek male patients with OSAS.

	Young and middle age (*n* = 568, m ± SD)	Elderly (*n* = 129, m ± SD)	*P* value
AGE	47.62 ± 5.34	70.54 ± 4.22	<0.001**^+^**
BMI	33.31 ± 6.42	32.18 ± 4.78	NS
Neck circumference	44.93 ± 4.06	44.72 ± 3.41	NS
Daytime sleepiness (ESS)*	11.21 ± 2.81	10.89 ± 2.62	NS
Cardiac arrhythmias**	7%	11%	<0.001**^+^**
Fatigue**	65%	60%	NS
Headache**	14%	14%	NS
Inability to concentrate**	2%	1.5%	NS

AGE: in years. BMI: body mass index (Kgr/m^2^), neck circumference, measured in cm. ^+^ *P* < 0.05, which was considered significant, *ESS: Epworth Sleepiness Scale (expressed as mean ± SD), **data are presented as %, NS: not significant.

**Table 2:** Polysomnographic findings in Greek male patients with OSAS.

	Young and middle age (*n* = 568, m ± SD)	Elderly (*n* = 129, m ± SD)	*P* value
AHI (events per hour)	35.14 ± 13.26	33.14 ± 12.09	NS
AHI-REM (events per hour)	30.72 ± 9.32	28.16 ± 7.89	NS
AHI-NREM (events per hour)	33.82 ± 12.14	31.73 ± 11.09	NS
Mean apnea/hypopnea duration (sec)	20.12 ± 6.12	22.37 ± 6.09	<0.001*
Hypoxemia duration in TST (min)	51.1 ± 19.12	73.50 ± 19.07	0.004*
Hypoxemia duration in REM sleep (min)	8.88 ± 2.46	7.98 ± 2.68	NS
Hypoxemia duration in NREM sleep (min)	28.08 ± 5.43	42.43 ± 6.98	0.021*
SatO_2_-REM	91.89 ± 7.12	89.91 ± 7.02	0.013*
SatO_2_-NREM	92.64 ± 5.21	90.32 ± 5.11	0.027*

AHI: apnea/hypopnea index, AHI-REM: apnea/hypopnea index in REM sleep, AHI-NREM: apnea/hypopnea index in NREM sleep, TST: total sleep time, Hypoxemia: time spent with SatO_2 _<90%, in minutes. SatO_2_-REM: oxyhemoglobin saturation in REM sleep. SatO_2_-NREM: oxyhemoglobin saturation in NREM sleep, NS: Not significant. *P < 0.05, which was considered significant.

**Table 3:** Correlation coefficients and results of stepwise multiple regression analysis between obesity-related variables and AHI.

	Young and middle age (*n* = 568)		Elderly (*n* = 129)	
	Correlation coefficient	Regression coefficient	Correlation coefficient	Regression coefficient
BMI	0.326 (*P* < 0.001*)	0.012 (*P* = 0.008*)	0.157 (*P* = 0.026*)	0.043 (*P* = 0.035*)
Neck circumference	0.380 (*P* < 0.001*)	0.368 (*P* < 0.001*)	0.245 (*P* < 0.001*)	0.227 (*P* = 0.001*)

BMI: body mass index. *P < 0.05, which was considered significant.
